# MicroRNA858-mediated regulation of anthocyanin biosynthesis in kiwifruit (*Actinidia arguta*) based on small RNA sequencing

**DOI:** 10.1371/journal.pone.0217480

**Published:** 2019-05-23

**Authors:** Yukuo Li, Wen Cui, Ran Wang, Miaomiao Lin, Yunpeng Zhong, Leiming Sun, Xiujuan Qi, Jinbao Fang

**Affiliations:** Zhengzhou Fruit Research Institute, Chinese Academy of Agricultural Sciences, Zhengzhou, P.R. China; Michigan State University, UNITED STATES

## Abstract

As important regulators, miRNAs could play pivotal roles in regulation of fruit coloring. *Actinidia arguta* is a newly emerged fruit tree with extensively application prospects. However, miRNAs involved in *A*. *arguta* fruit coloring are unknown. In this study, *A*. *arguta* fruit were investigated at three developmental stages by small RNAs high-throughput sequencing. A total of 482 conserved miRNAs corresponding to 526 pre-miRNAs and 581 novel miRNAs corresponding to 619 pre-miRNAs were grouped into 46 miRNA families. Target gene prediction and analysis revealed that miR858, a strongly candidate miRNA, was involved in anthocyanin biosynthesis in which contributes to fruit coloring. The anthocyanin level was determined in three *A*. *arguta* cultivars by UPLC-MS/MS (ultra-performance liquid chromatography coupled with tandem mass spectrometry). In addition, qPCR (quantitative real-time PCR), cluster analysis were conducted as well as correlation analysis. All results were combined to propose a model in which describes an association of miRNA and anthocyanin biosynthesis in *A*. *arguta*. The data presented herein is the first report on miRNA profile analysis in *A*. *arguta*, which can provide valuable information for further research into the regulation of the miRNAs in anthocyanin biosynthesis and fruit coloring.

## Introduction

Domestication of kiwifruit (genus *Actinidia*, *Actinidiaceae*) began in the twentieth century [1]. It is known as the ‘king of fruits’ as it contains high contents of amino acids, mineral components, antioxidants, vitamin C and abundant dietary fiber [2]. Kiwifruit belongs to the genus of *Actinidia* and comprises approximately 54 species and 125 taxa [3]. Commercial cultivars are mainly selected from two species: *Actinidia chinensis* and *Actinidia deliciosa* due to their unique taste, large-fruit and long-storage [4]. Kiwifruit is traditionally known as a green-fleshed fruit for a long time until the release of yellow-fleshed kiwifruit. Recently, an all-red-fleshed kiwifruit (*A*. *arguta*) which appears in the red skin and the flesh is introduced to the market. Consumers are attacted by its unique desirable agro characteristics such as appearance and high level of anthocyanin.

Anthocyanins, compose a group of flavonoids that are secondary metabolites and play important roles in the environmental adaptation [5], fruit development [6, 7], and even human health [8–11]. Anthocyanins are synthesized from a branch of flavonoids that also led to the synthesis of flavonols and proanthocyanidins [12]. The anthocyanin biosynthetic pathway has been extensively studied, and the majority of the structural and regulatory genes involved in anthocyanin accumulation have been isolated and identified in many model plants [13–23]and fruit species [24, 25]. The specific flow chart of anthocyanin biosynthetic pathway was also clearly drawn [26]. However, it is unclear what roles microRNAs (miRNAs) play in this process. Related studies were mainly focused on model plants, such as *Arabidopsis thaliana* [27] and *Solanum lycopersicum* [28], rarely in fruit trees, even less for kiwifruit.

As genome-encoded noncoding RNAs whose size ranges from approximately 18–24 nucleotides (nt), miRNAs play a crucial role in negatively regulating the expression of target genes via cleavage of complementary mRNAs or suppressing translation at the post-transcriptional level in eukaryotes [29, 30]. Plant miRNAs exert critical influence on the regulation of various biological processes, including development, primary and secondary metabolism and stress responses [30–33]. In recent years, several studies showed that miRNAs are involved in anthocyanin biosynthesis in *Arobidopsis*.The miR156 is a positve regulator which can repress the expression of *SPL9*, destabilizing the MBW transcriptional activation complex and thus preventing the expression of anthocyanin biosynthesis genes [32]. The miR408 is also a positive regulator, the over-expressed miR408 can increase anthocyanin accumulation in *Arabidopsis* seedlings [34, 35].The miR858a positively regulates anthocyanin biosynthesis by repressing the *MYBL2* in *Arabidopsis* seedlings [27]. In contrast, miR828 or TAS4-siRNA81 (-) negatively regulate anthocyanin biosynthesis in *Arabidopsis* [36–38]. The miR858 negatively regulates anthocyanin biosynthesis in tomato (*Solanum lycopersicum*) [28]. However, the majority of studies investigating anthocyanin biosynthesis are concentrated in model plants, only a few studies investigated miRNAs in kiwifruit. The miR172 influences kiwifruit flowering by interacting with the floral gene *AP2* [39]. However, no study has investigated the involvement of miRNAs in anthocyanin biosynthesis of *A*. *arguta*.

To investigate the miRNAs regulated molecular mechanism controlling fruit coloring in *A*. *arguta*, the all-red-fleshed *A*. *arguta* cultivar ‘Hongbaoshixing’ (‘HB’, an all-red-fleshed tetraploid kiwifruit cultivar by wild selection.) was selected as an experimental material for small RNA high-throughput sequencing. In addition, three *A*. *arguta* cultivars ‘HB’, ‘Rubysihao’ (RB-4, an all-red-fleshed tetraploid kiwifruit) and ‘Huanyouyihao’ (‘HY-1’, an all-green-fleshed tetraploid kiwifruit) were used for anthocyanin measurement. Based on our data presented in this study, a regulatory model was established to show the association of miRNAs and anthocyanin biosynthesis in *A*. *arguta*. These findings provide evidence that the miRNAs play a regulatory role in the coloring process in *A*. *arguta* and also expand our knowledge in understanding the regulation mechanisms of anthocyanin biosynthesis.

## Materials and methods

### Fruit materials

**Fig 1:**
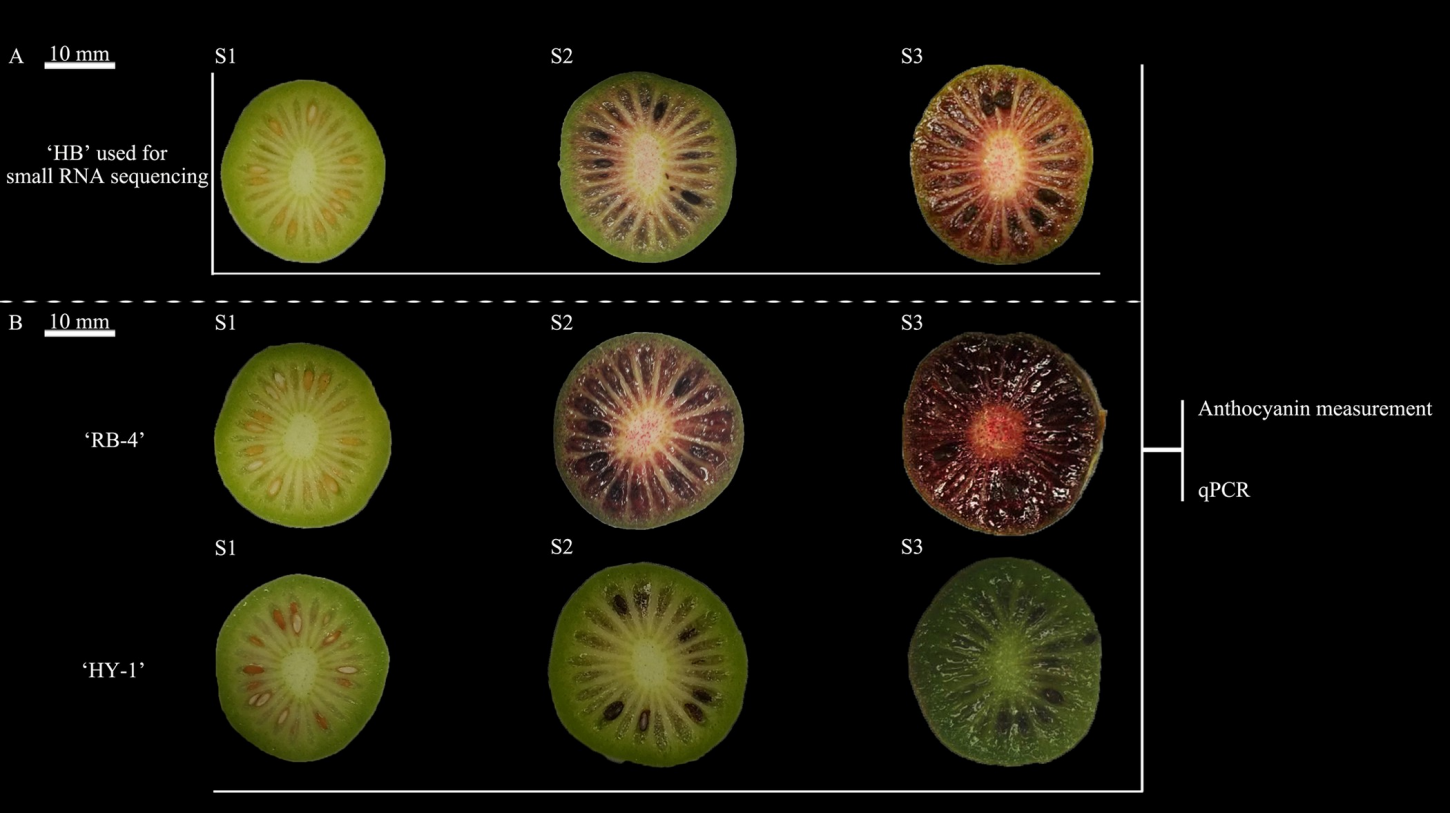
Change in fruit color at three stages: S1, S2 and S3. (A) Color change in all-red-fleshed kiwifruit ‘HB’. (B) Color change in all-red-fleshed kiwifruit ‘RB-4’ and all-green-fleshed kiwifruit ‘HY-1’. ‘HB’ was used for small RNA sequencing. ‘HB’, ‘RB-4’ and ‘HY-1’ were used for anthocyanin measurement and qPCR.

All materials are maintained at the National Kiwifruit Germplasm Garden of the Zhengzhou Fruit Research Institute, Chinese Academy of Agricultural Sciences, Henan Province, China. For small RNA sequencing, the fruits of the all-red-fleshed *A*. *arguta* cultivar ‘HB’ was harvested at three developmental stages, the dates of harvesting were recorded as days after full bloom (DAFB), including the S1 stage, at 70 DAFB (green fruit); the S2 stage, at 100 DAFB (the color-break stage, beginning to turn red); and the S3 stage, at 125 DAFB (ripe red fruit and also the stage of harvest maturity) (Fig 1A). In each stage, fruit samples used for small RNA sequencing were randomly collected from six different trees, every two of which served as a biological replication; thus, three independent biological replicates were included throughout the sequencing process. For anthocyanin content analysis and gene expression profiling, the fruits of ‘HB’ were harvested at different stages. ‘RB-4’ (an all-red-fleshed *A*. *arguta* kiwifruit cultivar) and ‘HY-1’ (an all-green-fleshed kiwifruit cultivar) were also included for this analysis (Fig 1B). The fruit was dissected using a blade, frozen immediately in liquid nitrogen, and then stored at -80°C until further use.

### Total RNA isolation, small RNA library construction, and sequencing

The total RNA was extracted using Trizol reagent (Invitrogen, CA, USA) in accordance with the manufacturer’s procedure. The total RNA quantity and purity were analyzed using a 2100 Bioanalyzer and an RNA 6000 Nano LabChip Kit (Agilent, CA, USA) with an RNA integrity number (RIN) >7.0. Approximately 1 μg of the total RNA was used to prepare small RNA libraries in accordance with the protocol of a TruSeq Small RNA Sample Prep Kit (Illumina, San Diego, USA). In addition, single-end sequencing (36 bp) was performed on an Illumina Hiseq 2500 at LC-BIO Company, Hangzhou, China. All sequencing was performed in triplicate.

### Data processing

The raw data reads were subjected to an Illumina pipeline filter (Solexa 0.3), and after which the dataset was further processed with an in-house program, ACGT101-miR (LC Sciences, Houston, Texas, USA) to remove adapter dimers, junk, low complexity, common RNA families (rRNA, tRNA, snRNA, and snoRNA) and repeats. Those unique sequences with a length in 18–25 nt were subsequently mapped to specific species precursors using miRBase 20.0 (http://www.mirbase.org/) by BLAST queries to identify both known miRNAs and novel 3p- and 5p-derived miRNAs. Variation in length at both 3’ and 5’ ends and one mismatch within the sequence were allowed in the alignment. Those unique sequences that mapped to the hairpin arms of the mature miRNAs of specific species were identified as known miRNAs; those unique sequences that mapped to the other hairpin arms of the precursors of known specific species (opposite that of the annotated mature miRNA-containing arm) were considered novel 5p- or 3p-derived miRNA candidates. The remaining sequences were mapped to other selected species precursors (excluding specific species) in miRBase 20.0 by BLAST queries, and to determine their genomic locations, the mapped pre-miRNAs were further BLASTed against the genomes of specific species. We defined the above two miRNA sequences as known miRNAs. The unmapped sequences were BLASTed against the specific genomes, and the RNA hairpin structures containing sequences were predicted from the 120 nt flanking sequences using RNAfold software (http://rna.tbi.univie.ac.at/cgi-bin/RNAfold.cgi).

### Quality control of biologically repeated samples and sequencing data

For biologically repeated samples, correlations of gene expression levels between different samples constitute an important indicator for testing both the reliability of experiments and the reasonability of sample selection; hence, correlation analyses between every two samples were conducted using Person correlation coefficients that were calculated using R Language [40]. In addition, principal component analysis (PCA) was used to examine the distribution of data and verify the reasonability of the experimental design. Finally, all data were submitted to the database NCBI Sequence Read Archive (SRA accession: PRJNA515826).

### Analysis of differentially expressed miRNAs

To determine the expression levels of miRNAs, the differential expression of miRNAs was analyzed by Chi square (N×N) tests; this analysis was performed on the basis of normalized deep-sequencing counts that were normalized as transcripts per million (TPM) using the formula normalized expression = mapped read count/total reads*10^6^ [41]. All differential expression between two samples was analyzed using the DEGseq R package [42]; the significance threshold for each test was set to be 0.01.

### Target gene prediction and enrichment analysis

Based on target penalty strategy, TargetFinder software was used to predict the putative target genes of miRNAs identified in this study [43]. In conjunction with the Gene Ontology (GO) and Kyoto Encyclopedia of Genes and Genomes (KEGG) information on kiwifruit (*A*. *chinensis*), GO functional annotation and KEGG signal pathway annotation for the differentially expressed miRNAs were carried out. All GO and KEGG pathway annotation for the predicted target genes of the differentially expressed miRNAs were subjected to Fisher’s accurate hypothesis tests, and, every GO and KEGG annotation was subjected to an enrichment analysis [44, 45].

### Measurements of anthocyanin contents

For ‘HB’, ‘RB-4’and ‘HY-1’, 10 g of flesh tissue per sample was ground and then extracted in a solution consisting of anhydrous ethanol, hydrochloric acid and water (volume ratio-2:1:1) twice by ultrasonic techniques. The anthocyanin components were qualitatively and quantitatively analyzed using UPLC-MS/MS (ultra-performance liquid chromatography coupled with tandem mass spectrometry) (Agilent, Palo Alto, CA, USA). Cyanidin (CAS: 528-58-5; chromatographic purity >98%; Chromadex), delphinidin (CAS: 528-53-0; chromatographic purity >96%), cyanidin-3-O-galactoside (CAS: 27661-36-5; chromatographic purity >98%; Chromadex) and delphinidin-3-O-galactoside (CAS: 28500-00-7; chromatographic purity >95%; Chromadex) were used as authentic standard samples for constructing a standard curve and for single point quantitation. Total anthocyanin content was measured using Plant Anthocyanin Content Detection Kit (Solarbio, Beijing, China) following manufacture’s recommendations. Each sample was analyzed in triplicate.

### Expression analysis by qPCR (quantitative real-time PCR)

The total RNA was isolated from the flesh samples of the three different cultivars at three different stages using the modified cetyltrimethyl ammonium bromide (CTAB) method [46]. One microgram of total RNA was used for cDNA synthesis with a RevertAid First Strand cDNA Synthesis Kit (Thermo Fisher Scientific, MA, USA). The reaction mixture and qPCR program used were in accordance with those described previously [47]. All analyses were repeated three times using biological replicates. In addition, the relative expression levels were calculated using the 2^-ΔΔCt^ method [48].

### Expression profiling of miR858 in red-fleshed and green-fleshed kiwifruit

The total RNA extraction of red-fleshed ‘HB’ and green-fleshed ‘HY-1’ at three developmental stages was carried out according to the Quick RNA Isolation Kit (Huayueyang Biotech, Beijing, China). Two hundred nanogram of total RNA was used for cDNA synthesis with TransScript miRNA First-Strand cDNA Synthesis SuperMix Kit (TransGen Biotech, Beijing, China). Quantitative real-time PCR was carried out using LightCycler 480 System (Roche Diagnostics). Thermal cycling conditions were 94°Cfor 30 sec, then 45 cycles of 94°C for 5 sec, 60°C for 15 sec and 72°C for 10 sec, which was described as 3 step protocol in TransStrat Tip Green qPCR SuperMix Kit (TransGen Biotech, Beijing, China). The 5S rRNA [27] was considered as the control gene for normalization. Three replicates were carried out for each analysis. 2^-ΔΔCt^ method was used to calculate relative expression level [48].

### Statistical analysis

Statistical analyses were performed using GraphPad Prism 5 (GraphPad Software Inc., San Diego, CA, USA), and cluster analyses were performed using both R-3.4.2 and TMEV software [49]. IBM SPSS Statistics 20 was used to test significant differences. Each value represents mean ± SD of three independent biological replicates.

## Results

### Quality control and overview of deep sequencing of small RNA

**Fig 2:**
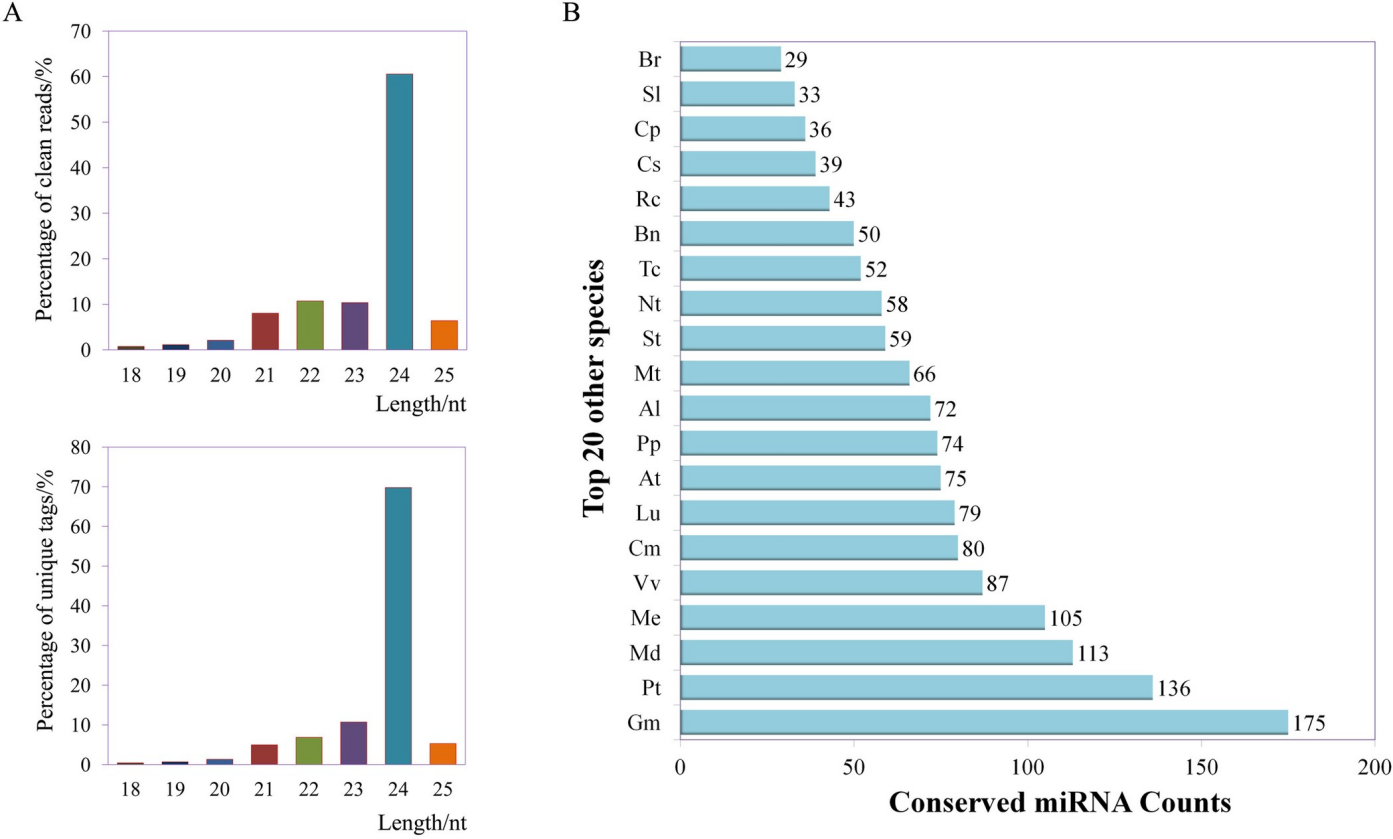
Analysis of sequence length and conservation of miRNAs. (A)Distribution of small RNAs with different sequence length according to clean reads and unique tags. (B) Conservation of the identified miRNAs with those in other species. The x-axis represents the number of conserved miRNAs and the y-axis indicates the top 20 number in other species. Different prefixes indicating different species are as follows: Br-*Brassica rapa*; Sl-*Solanum lycopersicum*; Cp-*Carica papaya*; Cs-*Citrus sinensis*; Rc-*Ricinus communis*; Bn-*Brassica napus*; Tc-*Theobroma cacao*; Nt-*Nicotiana tabacum*; St-*Solanum tuberosum*; Mt-*Medicago truncatula*; Al-*Arabidopsis lyrata*; Pp-*Prunus persica*; At-*Arabidopsis thaliana*; Lu-*Linum usitatissimum*; Cm-*Cucumis melo*; Vv-*Vitis vinifera*; Me-*Manihot esculenta*; Md-*Malus domestica*; Pt-*Populus trichocarpa*; Gm-*Glycine max*.

The ‘HB’ kiwifruit at three developmental stages was used for small RNA high-throughput sequencing. To ensure the reliability of the sequencing results, strict quality control was applied in this study. Person correlations and PCA between different samples verified the reliability of the data and the reasonability of the experimental design (S1 and S2 Figs). To identify possible miRNAs involved in the fruit development of ‘HB’, three independent small RNA libraries from ‘HB’ kiwifruit at three developmental stages were constructed and sequenced by high-throughput Illumina Solexa system. A total of 15,373,860 raw reads were obtained by sequencing. After removing the 3’ adapter sequences, reads whose length was < 18 nt and consecutive nucleotide dimers and trimer, 11,193,696 clean reads represented by 7,364,610 unique tags were selected (Table 1). The length distribution of clean reads and unique tags showed that the majority (96.0% for clean reads, 97.6% for unique tags) were 21–25 nt and the predominant sequences were 24 (60.5% for clean reads, 69.8% for unique tags) nt in length, followed by 23 nt and 22 nt (Fig 2A).This is consistent with the typical characteristics of Dicer enzyme cleavage.

**Table 1:** Overview of reads from raw data to cleaned sequences.

Type	Count	Percentage/%
Raw reads	15,373,860	100.000
3ADT&length filter	3,113,309	20.332
Junk reads	125,613	0.806
Rfam	528,255	3.484
mRNA	617,070	4.081
Repeats	7,735	0.052
Clean reads	11,193,696	72.654

Note: 3ADT&length filter: reads removed due to 3ADT not found and length with <18 nt and >25 nt were removed. Junk reads: Junk: > = 2N, > = 7A, > = 8C, > = 6G, > = 7T, > = 10Dimer, > = 6Trimer, or > = 5Tetramer. Rfam: Collection of many common non-coding RNA families except microRNA; http://rfam.janelia.org. Repeats: Prototypic sequences representing repetitive DNA from different eukaryotic species; http://www.girinst.org/repbase. Notes: valid reads may not be equal to raw reads - 3ADT&length, filter, Junk reads, mRNA, Rfam, Repeats, because there are overlapped sequences between mRNA, Rfam and Repeats. mRNA_Database: http://bioinfo.bti.cornell.edu/cgi-bin/kiwi/blast.cgi

### MiRNAs identification

To identify the conservative and novel miRNAs in the ‘HB’ flesh, the small RNA sequences were mapped to other plant miRNAs in the miRBase database. A total of 482 conservative miRNAs corresponding to 526 pre-miRNAs and a total of 581 novel miRNAs corresponding to 619 pre-miRNAs were identified (Table 2). The majority of these miRNAs were highly homologous to those in other plants, including *Glycine max*, *Populus trichocarpa*, *Malus domestica*, *Manihot esculenta* and *Vitis vinifera*. One hundred seventy-five miRNAs were identified in the ‘HB’ that homologous to those in *Glycine max* (Fig 2B). The sequences similarity revealed that these miRNAs were grouped into 46 miRNA families (S1 Table). Of these 46 miRNA families, six miRNA families including miR156, miR166, miR167, miR172, miR396 and miR398, were conserved in 32, 30, 27, 29, 32 and 25 plant species, respectively. The majority of the miRNAs were not conserved and has been only identified in one species. With the exceptions of miR862, miR2592, miR5645, miR6476 and miR7122, only one member was found in the remaining miRNA families (S2 Table).

**Table 2:** Summary of conservative and novel miRNAs.

Groups	Type	Pre-miRNA	Unique miRNA
Gp1	Conservative	33	49
Gp2a		164	175
Gp2b		295	223
Gp3		34	35
	Total conservative	526	482
Gp4	Novel	619	581
	Total	1,145	1,063

Note: Gp1 indicates reads map to specific miRNAs/pre-miRNAs in miRbase and the pre-miRNAs further map to the genome & EST; Gp2a indicates reads map to selected miRNAs/pre-miRNAs in miRbase. The mapped pre-miRNAs do not map to the genome, but the reads (and of course the miRNAs of the pre-miRNAs) map to genome. The extended genome sequences from the genome loci may form hairpins; Gp2b indicates reads were mapped to miRNAs/pre-miRNAs of selected species in miRbase and the mapped pre-miRNAs were not further mapped to genome, but the reads (and of course the miRNAs of the pre-miRNAs) were mapped to genome. The extended genome sequences from the genome loci may not form hairpins. Gp3 indicates reads map to slected miRNAs/pre-miRNAs in miRbase. The mapped pre-miRNAs do not map to the genome, and the reads do not map to the genome. Gp4 indicates reads do not map to selected pre-miRNAs in miRbase. But the reads map to genome & the extended genome sequences from genome may form hairpins.

### Prediction and analysis of miRNA targets

**Fig 3:**
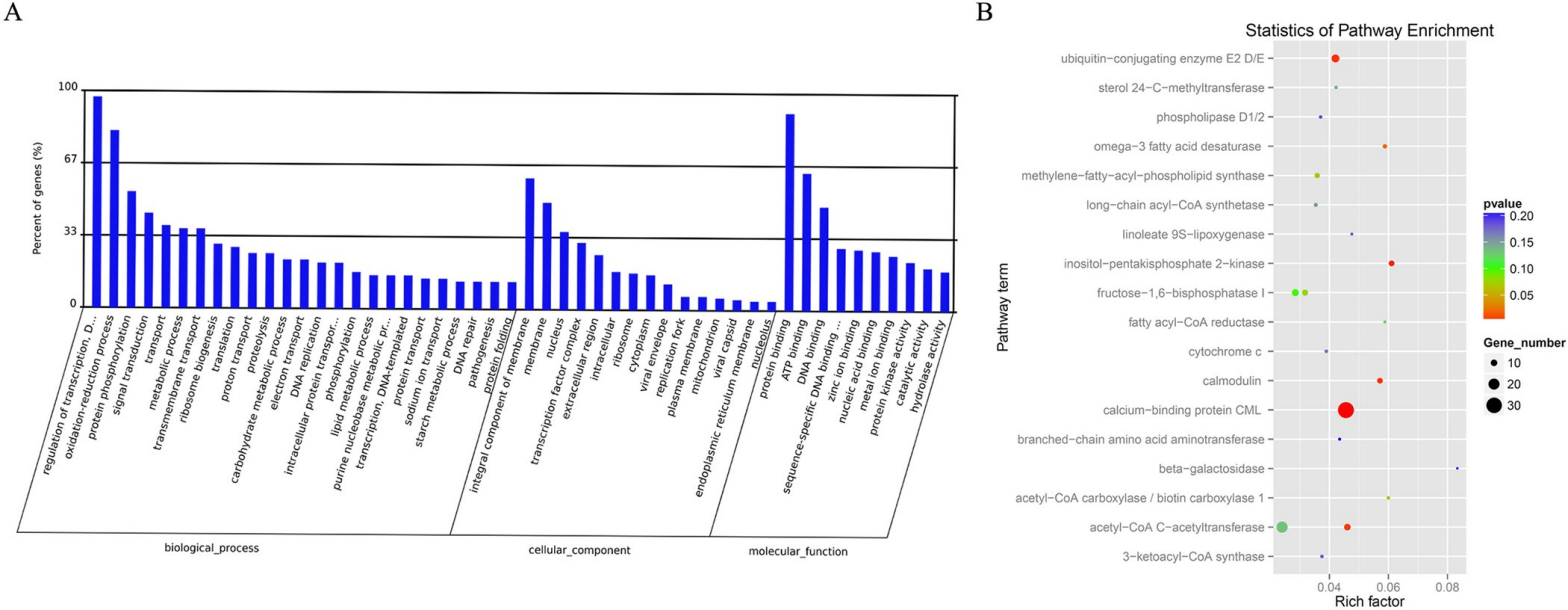
GO and KEGG analysis for miRNAs. (A) GO classification of target genes. Eight hundred fourteen target genes were assigned to three GO categories: 25, 15, and 10 GO terms were categorized as biological process, cellular components, and molecular functions, respectively. The x-axis represents the GO terms belonging to three GO categories, and the y-axis indicates the percent of genes. (B) KEGG pathway enrichment scatter plot. The horizontal axis represents the rich factor corresponding to the pathway, and the vertical axis represents the pathway name. P-values are represented by the color of the points. The number of genes with in each pathway is indicated by the size of point.

To better understand the biological function of the miRNAs obtained above, target genes for 321 identified miRNAs whose expression significantly differed (S3 Table) were predicted by TargetFinder software. The information of the predicted target genes included the miRNA ID, transcript ID, gene ID, score, range, strand, GO annotation and KEGG annotation (S4 Table). To obtain more detailed information about the function of the identified miRNAs, the predicted target genes were subjected to GO analysis (S5 Table). A total of 814 target genes were classified into three functional categories: biological processes, cellular components and molecular function, each of which contained 25, 15 and 10 GO terms, respectively (Fig 3A). Among those target genes, 129, 69 and 26 were assigned to ‘protein binding’ (GO: 0005515), ‘regulation of transcription’ (GO: 0006355) and ‘metabolic process’ (GO: 0008152), respectively. In addition, KEGG Pathway analysis was performed (S6 Table) and showed that 207 target genes were assigned into 18 pathway terms (Fig 3B), which were significantly related to response to ‘calcium-binding protein CML’ (ko04626) and ‘acetyl-CoA C-acetyltransferase’ (ko01200).

### Candidate miR858 involved in anthocyanin biosynthesis

**Fig 4:**
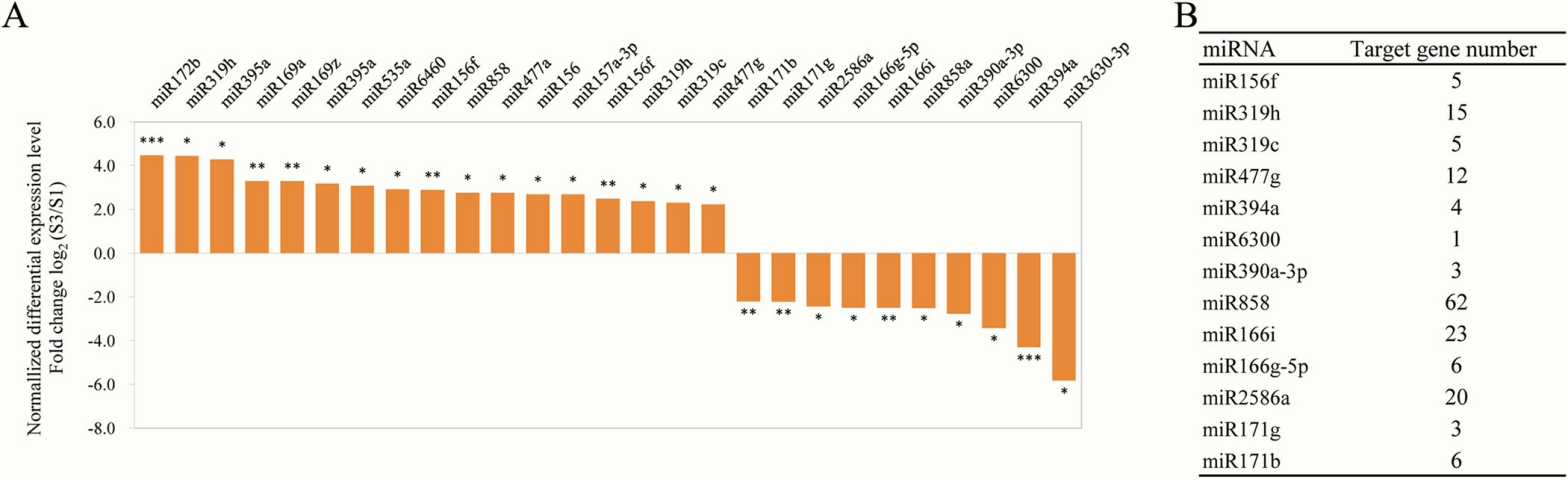
The miRNAs with significantly expressed difference and their target genes in comparsion S3 vs S1. (A) Up-regulated and down-regulated miRNAs, (B) Target gene number of 13 miRNAs. Statistical significance: *P<0.*05*, ** P<0.*01*, *** P<0.*001*.

Our study focuses on anthocyanins which can lead to fruit coloring. We are interested in identifying candidate miRNAs that contributes to the anthocyanin synthesis. Because of the phenotypic difference of ‘HB’ fruit color between S1 (fruit is green) and S3 (fruit is completely red) was the most obvious, so the comparison of S3 vs S1 was served as the cut-in spot for analysis. The results for conserved miRNAs with significantly expressed difference in comparison of S3 vs S1 showed that 17 conserved miRNAs were up-regualted and 10 conserved miRNAs were down-regulated, respectively (Fig 4A). Furthermore, the target genes of these miRNAs were also predicted and identified. The number of target genes for individual miRNAs varies from 1 to 62. For instance, miR858 tops the list with 62 target, followed by miR166i and miR2586a, which have 23 and 20 targets, respectively (Fig 4B). Among these targets, only c105731_g1 (named *AaMYBC1*) which is the target of miR858 was related with anthocyanin biosynthesis (S7 Table), which guided us to take miR858 as the object for next study.

### Analysis of anthocyanin components and content

**Fig 5:**
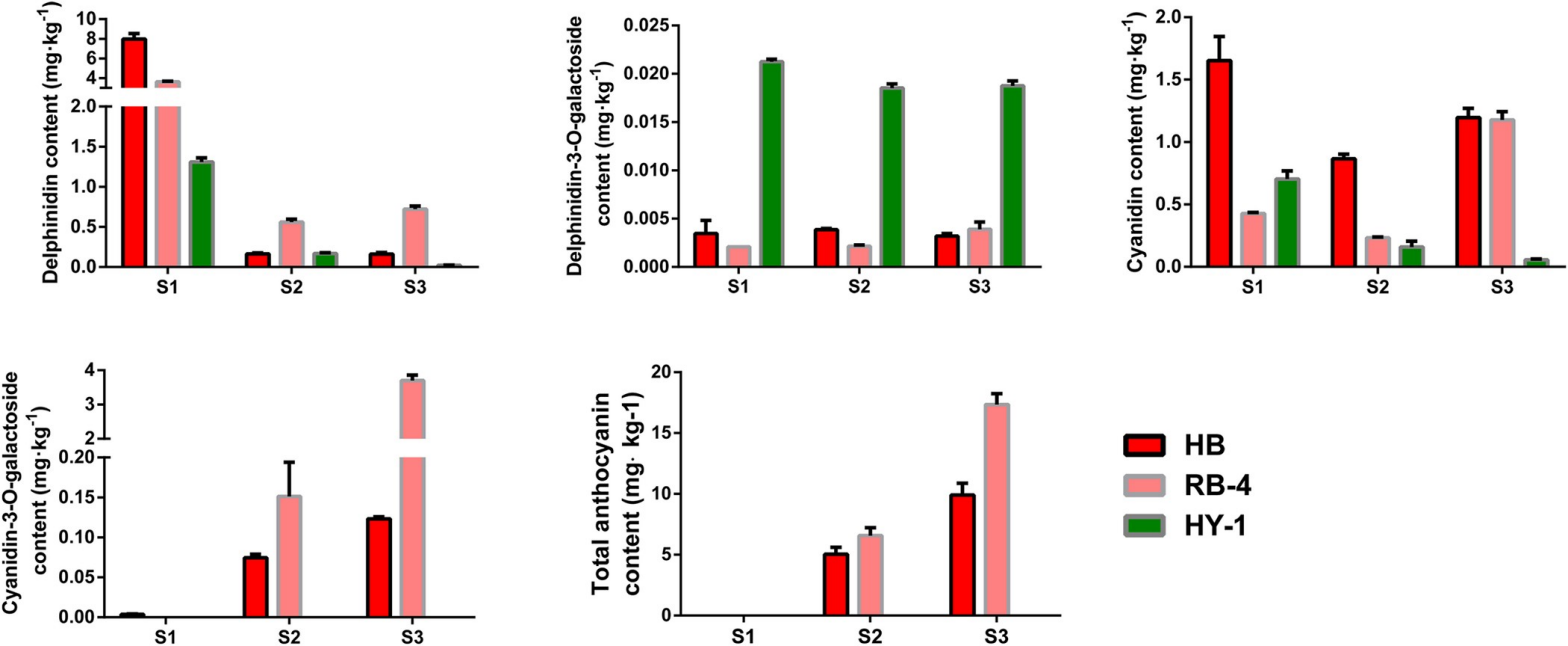
Changes of four different typed anthocyanin ant total anthocyanin content in the flesh of ‘HB’, ‘RB-4’ and ‘HY-1’ kiwifruit during three different developmental stages S1, S2 and S3. Data are means SD of three replicates. Red, pink and green column represent ‘HB’, ‘RB-4’ and ‘HY-1’, respectively.

To investigate the relationship between fruit color and anthocyanin components and content, three different *A*. *arguta* cultivars (‘HB’, ‘RB-4’and ‘HY-1’) were served as materials and four different anthocyanin types were served as standard for measuring anthocyanin components and content (S3 Fig). In the flesh tissues of the three *A*. *arguta* cultivars, these four anthocyanin components were detected in ‘HB’ and ‘RB-4’. Except for cyanidin-3-O-galactoside, the remaining three components were detected in ‘HY-1’, but the content was extremely low. Cyanidin and delphinidin content at S1 were higher than that at S3 in ‘HB’ and ‘RB-4’, whereas cyanidin-3-O- galactoside content was just the opposite (Fig 5), which indicate the cyanidin-3-O-galactoside was the main chromogenic pigment in *A*. *arguta* and also suggest that it is just because of the existence of cyanidin-3-O-galactoside leading to color differences between red- and green-fleshed *A*. *arguta* cultivars. The changing trend of total anthocyanin content at three stages was similar with that of cyanidin-3-O-galactoside.

### Gene expression profiles, cluster and correlation analysis

**Fig 6:**
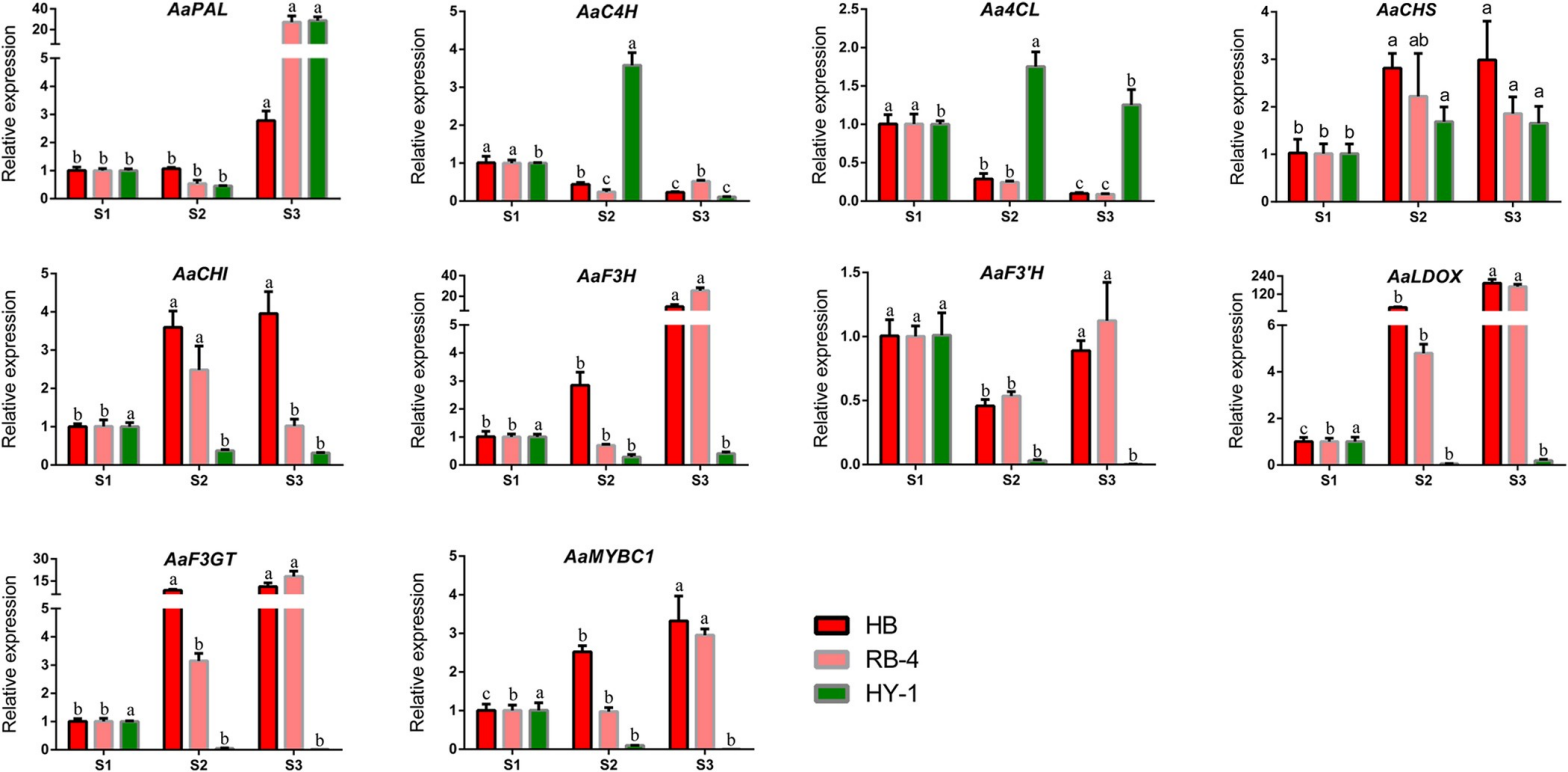
Expression profiles of anthocyanin pathway genes at 3 stages (S1, S2 and S3) in ‘HB’, ‘RB-4’, and ‘HY-1’ kiwifruit. The results represent the means SD of three replicates. Different lowercase lettters indicate significant difference at *P*≤ 0.05.

**Fig 7:**
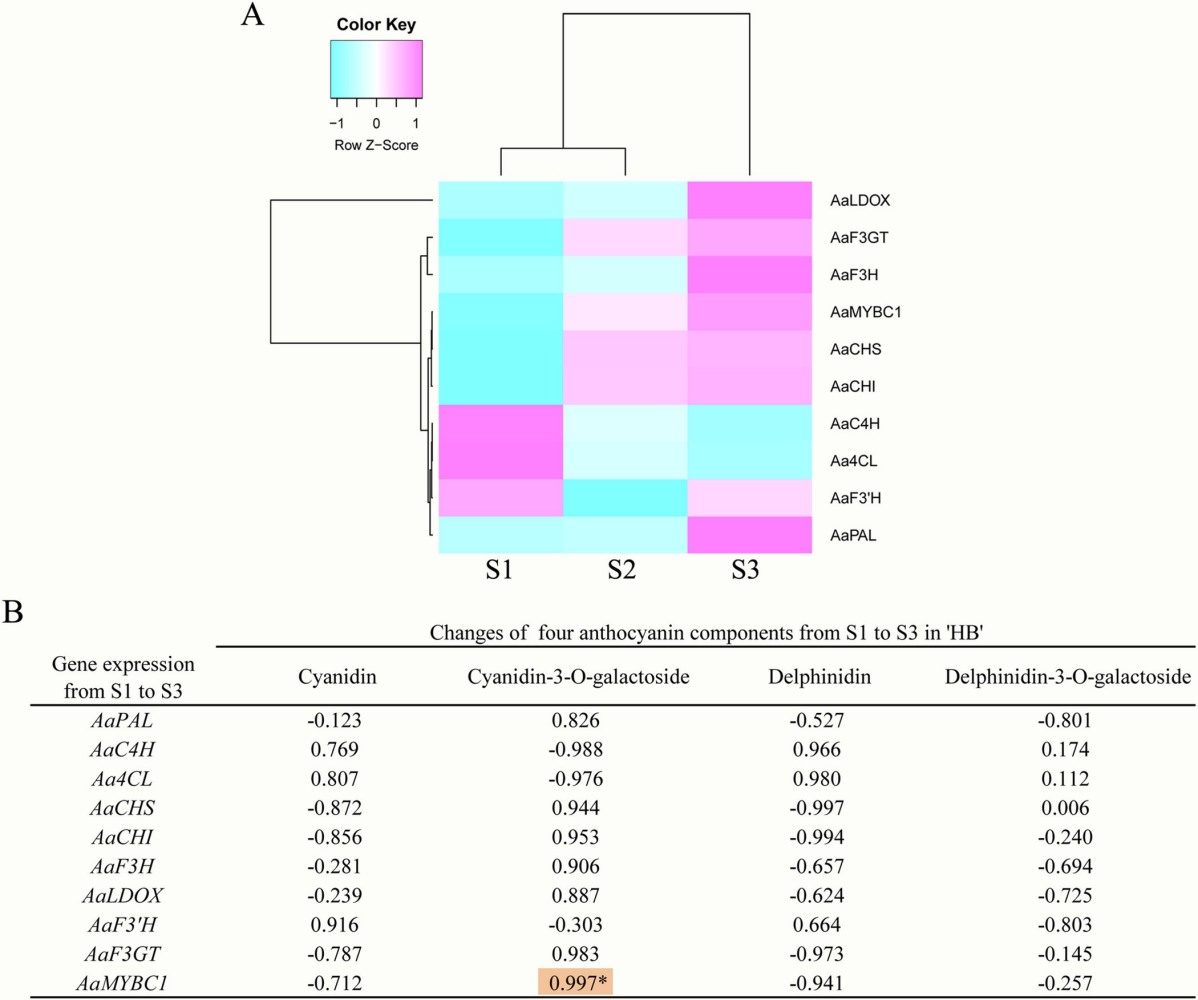
Cluster and correlation analysis. (A) Cluster analysis of gene expression in ‘HB’; (B) Correlation analysis between content of four anthocyanin components and gene expression from S1 to S3 in ‘HB’. The purple boxes indicate high expression levels, and the cyan boxes indicate low expression levels. ‘*’ indicates correlation is significant at 0.05 level.

**Fig 8:**
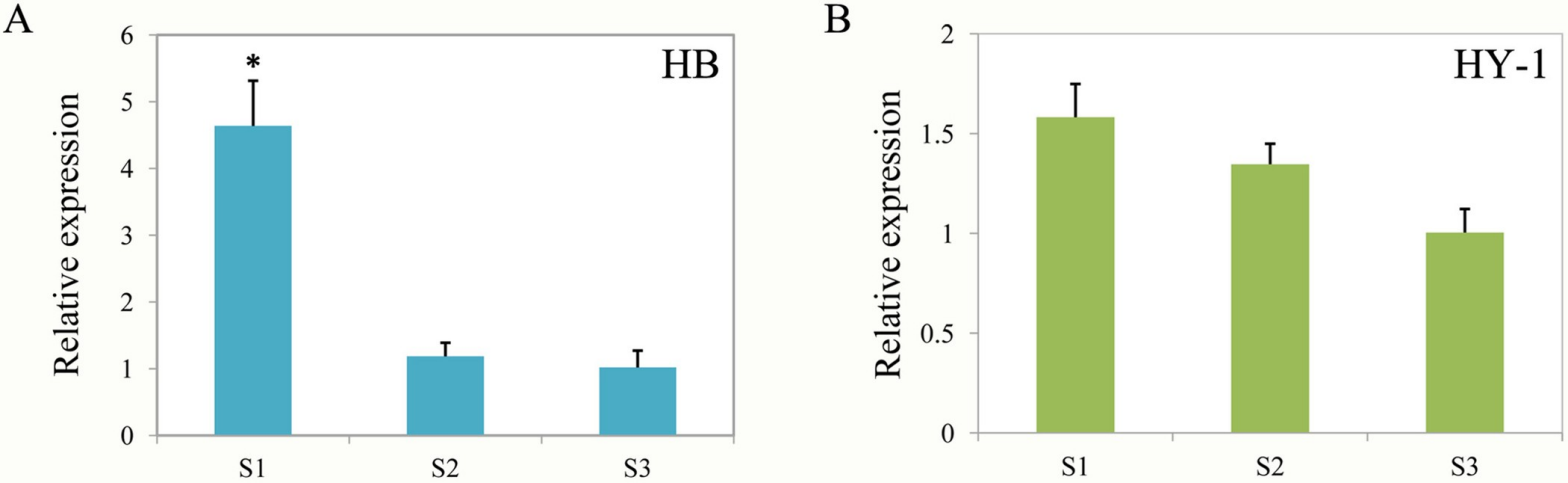
Expression comparsion of miR858 in red-fleshed ‘HB’ and green-fleshed ‘HY-1’ kiwifruit at three developmental stages. (A) Expression level of miR858 in ‘HB’. (B) Expression level of miR858 in ‘HY-1’. The Results represent the means SD of three replicates. Data were analyzed with Student’s t-test (*P < 0.05).

On the basis of our previous transcriptome results, we used the sequenced coding DNA sequence (CDS) of *AaMYBC1* as well as 8 structural genes involved in anthocyanin biosynthesis for designing primers and performing qPCR (S8 Table). The results clearly showed that the expression levels of three structural genes *(AaF3H*, *AaLDOX* and *AaF3GT)* tended to increase from S1 to S3 and peaked at S3 in the two red-fleshed *A*. *arguta* cultivars ‘HB’ and ‘RB-4’; however, the low expression trend of these genes was observed at S3 in the green-fleshed *A*. *arguta* cultivar contrasted with that in the red-fleshed ones. The same pattern was observed for *AaMYBC1* (Fig 6). The cluster analysis showed that the gene, *AaLDOX*, clustered into a single class in ‘HB’ (Fig 7A, S9 Table), which indicate that *AaLDOX* plays a key role in anthocyanin biosynthesis. Correlation analysis revealed that there was significant correlation between gene expression of *AaMYBC1* and cyanidin-3-O-galactoside content from S1 to S3 in ‘HB’ (Fig 7B, S10 Table). Together, these results suggested that *AaMYBC1* and *AaLDOX* played a key role in anthocyanin biosynthesis, which contributed to the fruit coloring in *A*. *arguta*. In addition, expression difference of miR858 between red-fleshed and green-fleshed kiwifruit showed that the expression level of miR858 was significant higher at S1 (green flesh) than S3 (red flesh) in ‘HB’ (Fig 8A), while there were no significant difference of miR858 expression at S1, S2 and S3 in ‘HY-1’ (Fig 8B), which validated the correctness of the sequencing results.

### The miRNA-target gene regulatory network of anthocyanin biosynthesis

**Fig 9:**
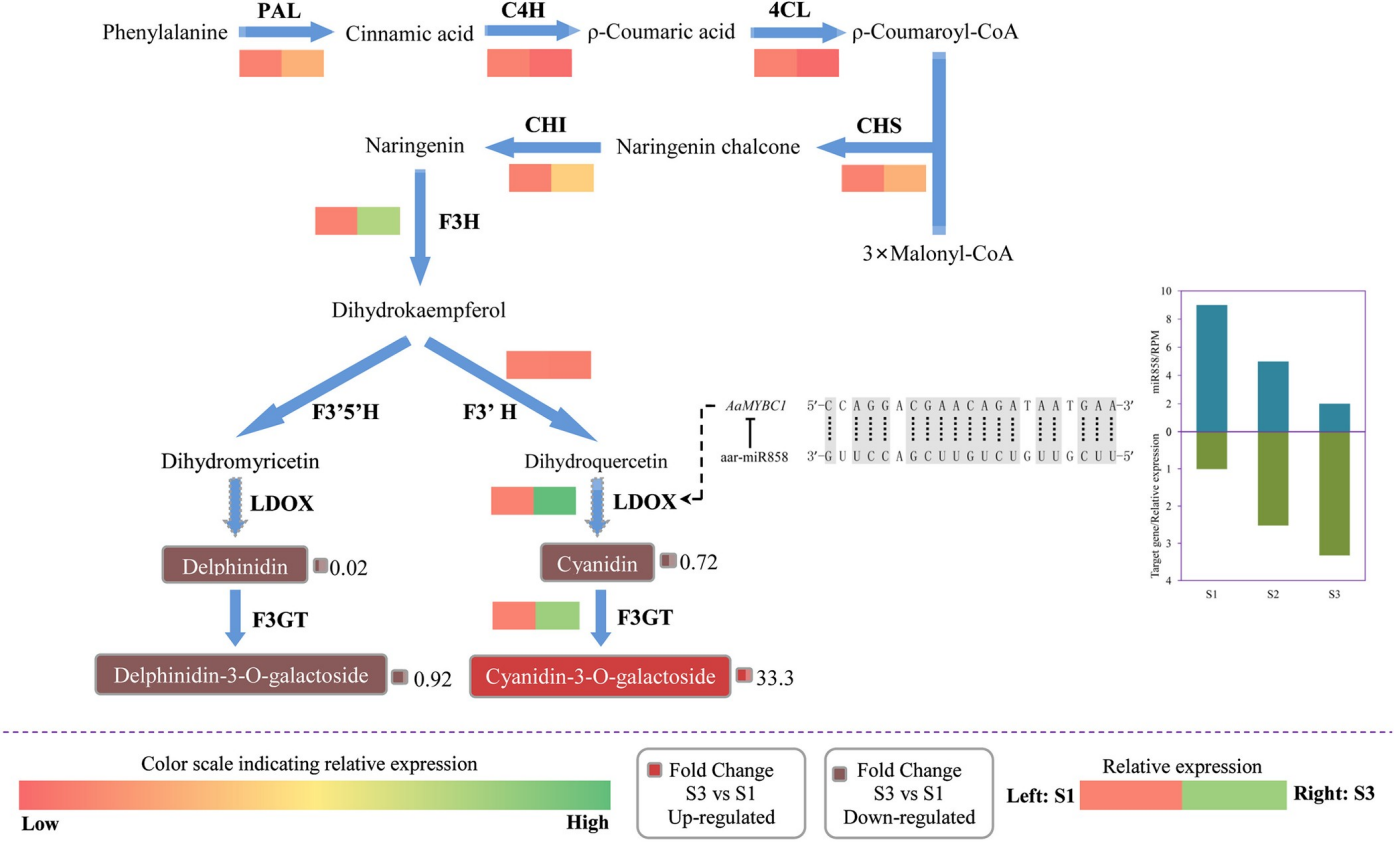
*Actinidia arguta* miRNA-target gene regulatory network involved in anthocyanin biosynthesis at two development stages S3 and S1. The right presentation showed the cooresponding miRNA:target alignment and the expression levels of miR858 and its target gene from S1 to S3. T bar refer to negative role of miRNA on its target gene. Dotted arrow indicates indirect regulation of miRNA on anthocyanin biosynthesis. Color scale from red to green represent relative expression level from low to high. Small red and dark red grid represent up-regulated and down-regulated fold change of different anthocyanin components between S3 and S1, respectively. PAL, phenylalanine ammonia-lyase; C4H, trans-cinnamate 4-hydroxylase; 4CL, 4-coumarate: CoA ligase; CHS, Chalcone synthase; CHI, Chalcone isomerase; F3H, flavanone 3-hydroxylase; F3’H, flavonoid 3'-hydroxylase; LDOX, leucoanthocyanidin dioxygenase; F3GT, flavonoid 3-O-glcosyl-transferase.

To better understand the role of miRNAs involved in anthocyanin biosynthesis, all results were combined to establish a network to show the relationship between miRNAs and anthocyanin biosynthesis (Fig 9). The miR858 was identified in the present study as well as its target genes *AaMYBC1* which encodes a transcription factor. The expression level of miR858 gradually decreased from S1 to S3, while their targets gradually increased from S1 to S3. The miRNA:target alignment showed that miR858 inhibit the expression of *AaMYBC1* by incomplete complement. Expression differences of *AaLDOX* regulated by transcription factor AaMYBC1 resulted in content differences of four anthocyanin components in *A*. *arguta*, which leads to distinct fruit color. The proposed network would provide a new insight for revealing the red mechanism of *A*. *arguta*.

## Discussion

MiRNAs are important regulators involved in various biological processes, including biotic and abiotic stress tolerance, plant growth and development, metabolic pathways and morphogenesis,. Extensive reports show that miRNAs regulate fruit development in multiple plants, including orange [50], pear [51], tomato [52], and persimmon [53]. Although many miRNAs have been identified by small RNA sequencing in both model plants and many fruit trees including *Arabidopsis thaliana* [54], tomato [55], rice [56], apple [57] and peach [58], miRNAs have not been characterized in *A*. *arguta*. The results of our previous study suggests that anthocyanin accumulation in *A*. *arguta* during fruit development is accompanied by the expression of relevant gene, indicating that fruit coloring is both a dynamical process and a complex network regulated by a series of genes [48, 59]. Therefore, the identification of miRNAs in *A*. *arguta* can provide valuable information to better understand the biological process in fruit coloring.

### MiRNAs were identified by small RNA sequencing

In this study, after removing any redundancy among 1,315 original miRNAs, we obtained 1,063 miRNAs, including 482 known miRNAs corresponding to 526 pre-miRNAs and 581 novel miRNAs corresponding to 619 pre-miRNAs, which were grouped into 46 miRNA families. (Table 2, S1 Table). In order to obtain functional information of miRNAs, the target genes of 321 identified miRNAs were identified and were used for further analysis of GO and KEGG pathway enrichment. A total of 814 and 207 target genes were assigned into three GO functional categories and 18 KEGG pathway terms (Fig3A, Fig 3B, S3–S6 Tables). To seek for miRNAs that are involved in anthocyanin synthesis, the comparison of S3 vs S1 was confirmed as cut-in spot for further analysis. Finally, we identified miR858 as a candidate miRNA and also found that its target gene *AaMYBC1* encoding a transcription factor is involved in anthocyanin biosynthesis.

### The content of four anthocyanin components

As important color pigments, anthocyanins are widely found in various plant species including kiwifruit. In this study, four different anthocyanin components and total anthocyanin content were measured in the flesh of three *A*. *arguta* cultivars at three developmental stages. The cyanidin-3-O-galactoside was detected in the red-fleshed *A*. *arguta* ‘HB’ and ‘RB-4’, but rarely in green-fleshed *A*. *arguta* ‘HY-1’ fruit (Fig 5). It indicates that the cyanidin-3-O-galactoside is the dominant chromogenic pigment in *A*. *arguta*, which contribute to color differences between red- and green-fleshed *A*. *arguta* cultivars. It provides evidence that this particular anthocyanin component plays a crucial role in fruit coloration in *A*. *arguta*. It is consistent with the results reported previous, in which the red color of the red-fleshed *A*. *arguta* is due to anthocyanin accumulation [47, 59–62].

### Key genes involved in anthocyanin biosynthesis

Gene expression is fundamental and indispensable to the integrity of biological processes. The spatio-temporal specificity of gene expression is deliberately controlled by transcription factors that are involved in sophisticated regulatory networks. In addition to transcription factors, the miRNAs and their target genes also exert considerable influence on the expression of downstream genes through modulating the regulatory network [63]. In plant species, extensive studies have suggested that anthocyanin biosynthesis is a sophisticated process which is regulated by multiple exogenous and endogenous factors, such as transcription factors [27]. Many transcription factors have been identified in kiwifruit. For example, *AcMYB110* is an R2R3 MYB that regulates the coloration of red petals in kiwifruit [64], and *AcMYBF110* is a crucial MYB that regulates anthocyanin biosynthesis in red kiwifruit [62]. In our study, AaMYBC1 transcription factor is found to play a crucial role in anthocyanin biosynthesis in *A*. *arguta*. This suggests that the key transcription factors involved in anthocyanin biosynthesis might be different in different *Actinidia* species. Structural genes encoding enzymes that participate in anthocyanin biosynthesis have been identified and cloned in kiwifruit [62, 65]. In our study, a structural gene *AaLDOX* was considered the key structural gene involved in anthocyanin biosynthesis in *A*. *arguta*, which is inconsistent with previous suggestions by which the formation of red inner pericarp of ‘HD22’ and ‘Hongyang’ results from high expression levels of *AcF3GT* and *UFGT*, respectively [62, 65]. This contradiction suggests that the structural genes regulating anthocyanin biosynthesis may differ among *Actinidia* species. The result of correlation analysis showed that expression of *AaMYBC1* at three stages was strongly correlated to anthocyanin content in *A*. *arguta* ‘HB’ (Fig 7), indicating that the *AaMYBC1* positively regulates anthocyanin biosynthesis, thereby we speculated that these two genes, *AaMYBC1* and *AaLDOX* play a key role in anthocyanin biosynthesis in *A*. *arguta*.

### Establishment of regulatory network model

In summary, the above mentioned results were combined to propose a model in which describes an association of miRNA and anthocyanin biosynthesis in *A*. *arguta* (Fig 9). When the fruit is green at S1, miR858 highly expresses and combines with it’s target gene *AaMYBC1* encoding AaMYBC1 transcription factor that can act on promoter of *AaLDOX* structural gene. This combination indirectly suppresses the transcription of *AaLDOX*, which encode key enzymes that participate in anthocyanin biosynthesis. This suppression explains why the expression levels of *AaMYBC1* and *AaLDOX* are extremely low. However, when the fruit is red at S3, the expression level of miR858 is low while its target gene is high, the anthocyanin biosynthesis normally recover. As negative regulator, miR858 suppresses anthocyanin biosynthesis in *A*. *arguta*, which agree Jia et al. [28], who suggested miR858 is a negative regulator of anthocyanin biosynthesis in tomato, but disagree with those of Wang et al. [27], who suggested that, by repressing the expression of *MYBL2*, miR858a positively regulate anthocyanin biosynthesis in *Arabidopsis* seedlings. These finding suggest that miRNAs may serve distinct functions across species.

## Supporting information

S1 Fig Pearson correlations between different samples. (TIF)

S2 Fig PLS-DA. (TIF)

S3 Fig UPLC profile results of different samples. (TIF)

S1 Table Relevant information on miRNA families. (XLSX)

S2 Table Relevant information on miRNAs in other species. (XLSX)

S3 Table Identified miRNAs whose expression significantly differ. (XLSX)

S4 Table Information on predicted target genes. (XLSX)

S5 Table GO analysis of target genes. (XLSX)

S6 Table KEGG pathway analysis of target genes. (XLSX)

S7 Table Annotation of target genes for 13 miRNAs. (XLSX)

S8 Table Primer sequences of genes used for qRT-PCR. (XLSX)

S9 Table Data used for cluster analysis. (XLSX)

S10 Table Gene expression and content of anthocyanin components from S1 to S3 in 'HB'. (XLSX)
