# Supplementary figures and images for: MicroRNA858-mediated regulation of anthocyanin biosynthesis in kiwifruit (Actinidia arguta) based on small RNA sequencing
Source: PLoS One. 2019 May 23;14(5):e0217480. doi: 10.1371/journal.pone.0217480 (PMC6532936; doi:10.1371/journal.pone.0217480)

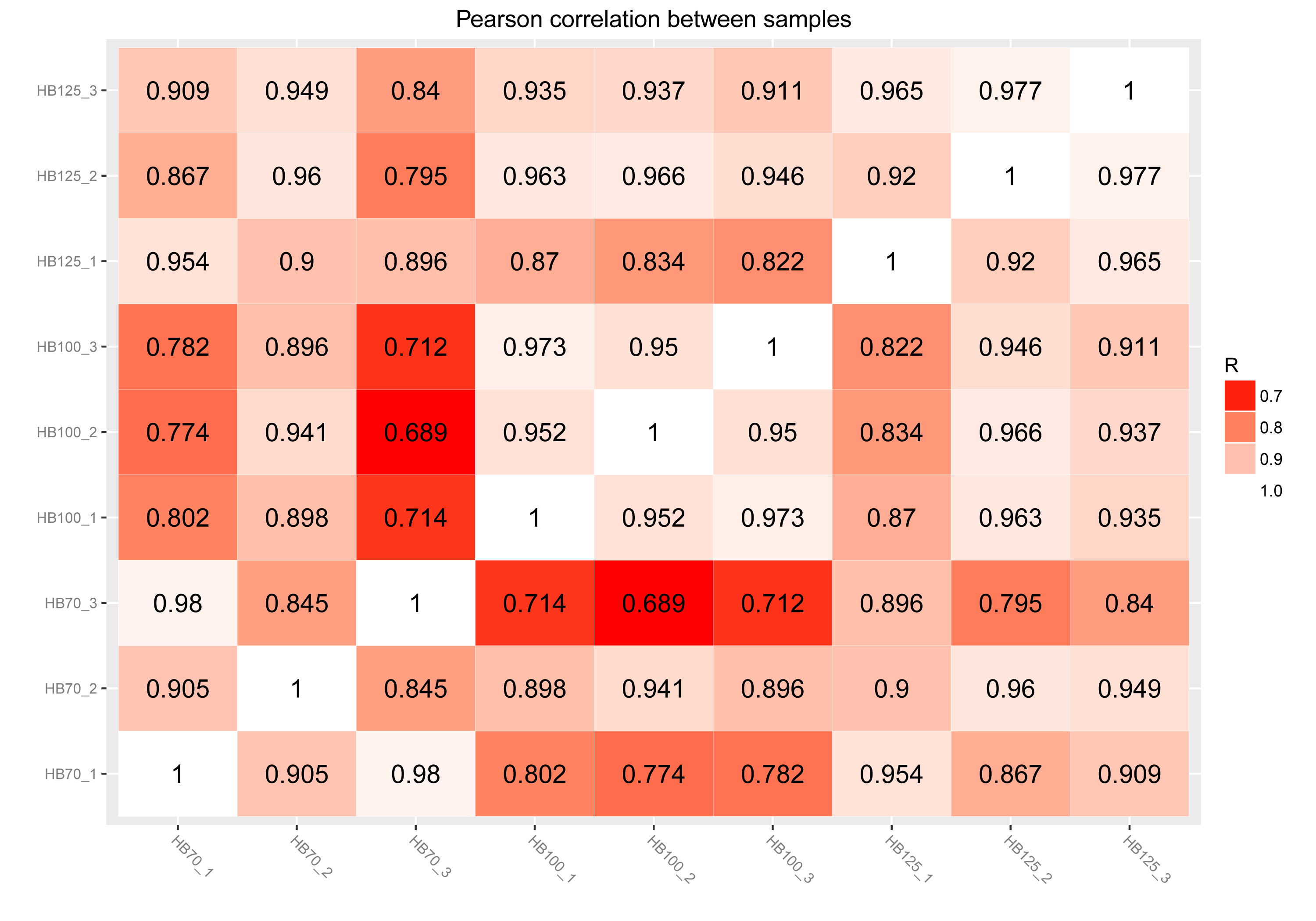

Supplement: S1 Fig — (TIF) [file pone.0217480.s001.TIF]

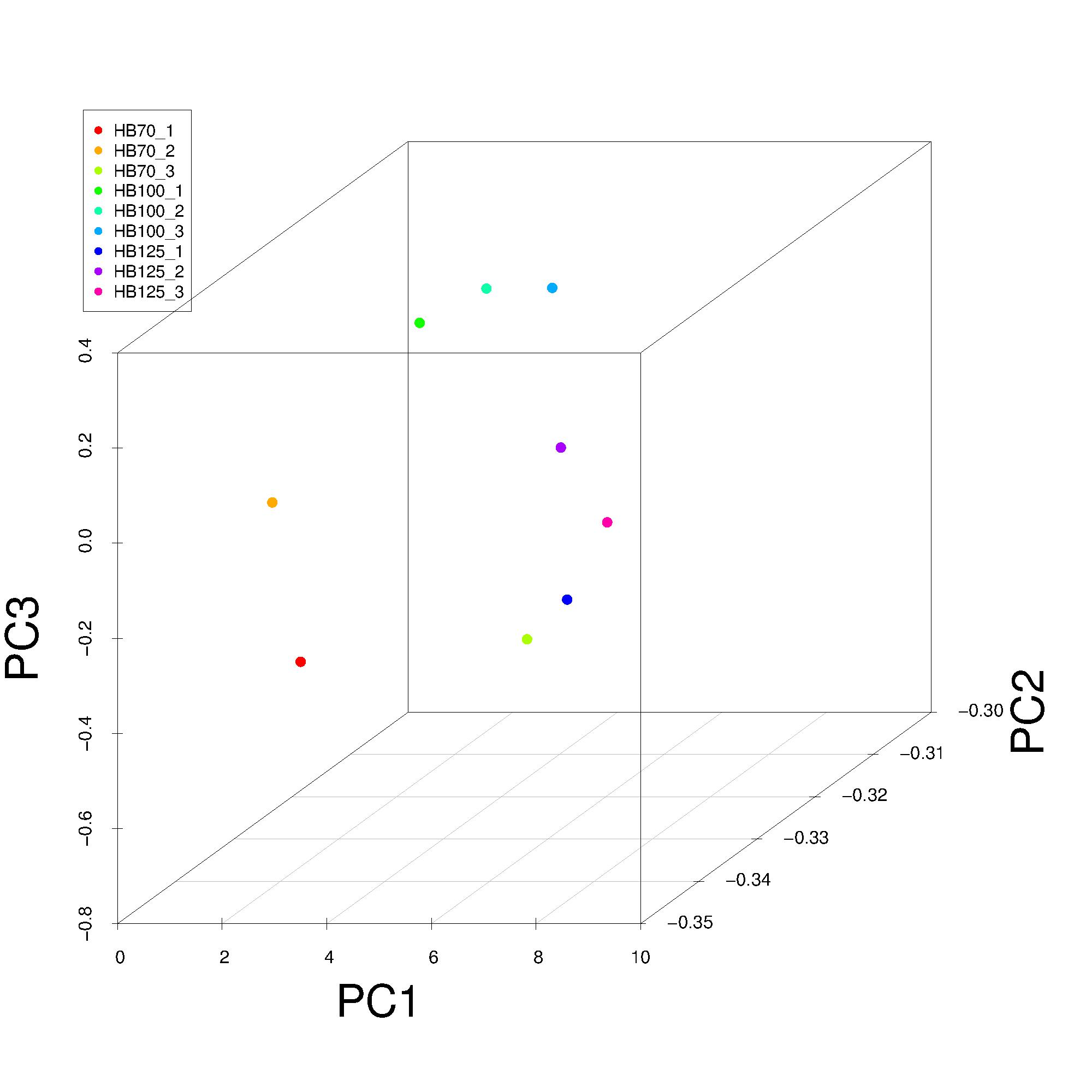

Supplement: S2 Fig — (TIF) [file pone.0217480.s002.TIF]

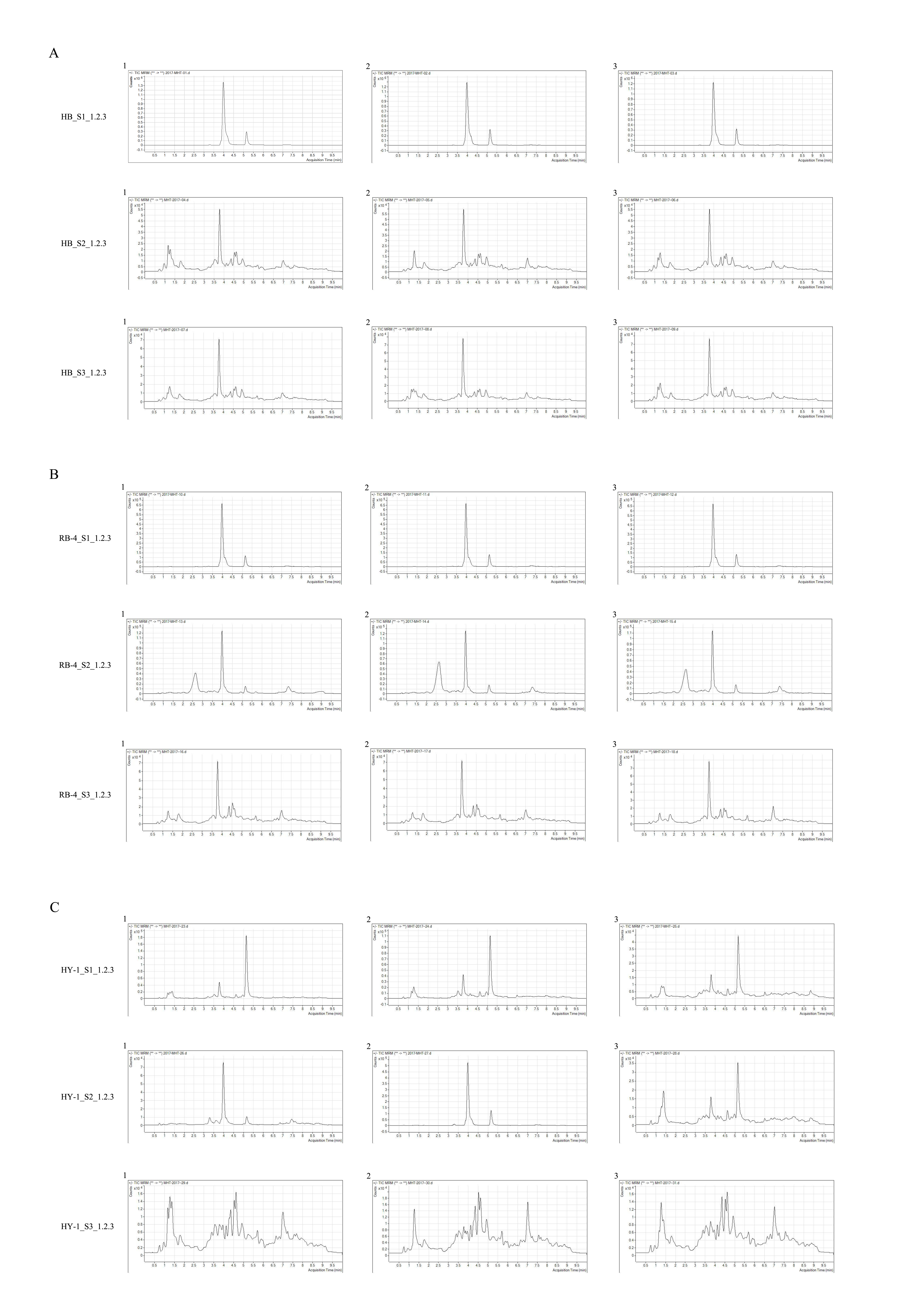

Supplement: S3 Fig — (TIF) [file pone.0217480.s003.TIF]
